# An Account of a Remarkable Nervous Affection

**Published:** 1782

**Authors:** 


### SECTION II.

### Essays and Observations.

I. *An Account of a remarkable nervous Affection. By Mr.* Charles Kite, *Surgeon at Graveſend in Kent. Communicated in a Letter to* Samuel Foart Simmons, M. D. F. R. S. *Read* Aug. 5th, 1782.

A Woman, aged 38, of a plethoric habit, was attacked in the year 1773 with an inflammation on her inſtep, which at firſt appeared like a ſmall pimple; but in a very ſhort time increaſed conſiderably.

It

It is unneceffary to take up the attention of the fociety with a relation of minute circumftances. It will, I prefume, be quite fufficient to obferve, that the inflammation fpread round the ancle and up to the knee. Ulceration took place, and the tibia was difcovered to be carious through almoft its whole length. It was from the beginning attended with the moft excruciating torture. Every remedy was made ufe of, which the fkill and experience of a very eminent furgeon could fuggeft. Bleedings occafionally repeated, emollient fomentations, and cataplafms prepared with decoction of poppies, bread, and opium, feemed to give moft eafe. Barbadoes tar, leeches, blifters, cupping, cauftics, rafping the bone, and falivation were tried; but without the leaft good confequence arifing, and very ftrong opiates ferved only to aggravate her miferies.

After fhe had endured the moft violent torments for the fpace of eleven or twelve months, it was deemed neceffary to amputate the limb above the knee. To this operation fhe very readily fubmitted, naturally concluding it would effectually remove her prefent agonies; but in this fhe was unhappily miftaken, for a few days after the leg was taken of, fhe was again attacked tacked with her old diforder, in a degree equally diftreffing and excruciating as before, and (as fhe imagined) occupying exactly the fame places; to wit, all the fpace between the ancle and the knee. It continued three or four days, and then went off. This the furgeons endeavoured to perfuade themfelves was no more than a common nervous fenfation, and what every one after amputation experiences in a greater or lefs degree. A very fhort time, however, fully convinced them, there was fomething more extraordinary in this cafe than what is ufually obferved in others; for in about fix weeks the fame painful fymptoms occurred again, and during the fpace of feven years generally returned once in about two months; but fhe has had only two fevere fits within thefe laft two years.

The pain is preceded by fhivering and ficknefs, after which the pulfe becomes rather more quick and full, and the tongue white. The ftump during this period is not in the leaft inflamed—It may even be preffed very hard with impunity; but when the pains are very ftrong, it is repeatedly jerked upward with fo violent a motion, that fhe cannot retain it in the ufual pofition with both her hands. She is always always relieved by bleeding, aperients, anodynes, and camphor; but notwithftanding this plan is followed on the firft appearance of the complaint, it is ufually three or four days, fometimes longer, before it entirely forfakes her.

Now and then fhe is fubject to very fevere pains in the ftomach and bowels; and at thefe times is always exempt from the pains, which fhe conceives to be in the leg; but fhe has remarked, that the pain in the extremity has frequently come on immediately after that in the ftomach has difappeared, and *vice verfa.*

Before the operation her menfes were obftructed, but ever fince they have been tolerably regular.

